# Ultrafast Photodissociation and Recombination Dynamics of Methyl *p*‑Tolyl Sulfoxide in Solution: Insights from Transient Absorption Spectroscopy and Computational Studies

**DOI:** 10.1021/acsomega.5c11932

**Published:** 2026-05-25

**Authors:** Alessandra Paladini, Patrick O’Keeffe, Francesco Toschi, Daniele Catone, Stefano Turchini, Giuseppe Ammirati, Pierluca Galloni, Mauro Satta, Susanna Piccirillo

**Affiliations:** † CNR-Istituto di Struttura della Materia (CNR-ISM), EuroFEL Support Laboratory (EFSL), Via Salaria km 29,300, 00015 Monterotondo Scalo, RM, Italy; ‡ CNR-Istituto di Struttura della Materia (CNR-ISM), 204549 EuroFEL Support Laboratory (EFSL), Via del Fosso del Cavaliere 100, 00133 Roma, Italy; § Dipartimento di Scienze e Tecnologie Chimiche, Università degli Studi di Roma “Tor Vergata”, Via della Ricerca Scientifica, 00133 Rome, Italy; ∥ CNR-Istituto per lo studio dei Materiali Nanostrutturati (CNR-ISMN), Dip.to di Chimica, Università Sapienza di Roma, P. le Aldo Moro 5, 00185 Roma, Italy

## Abstract

When molecular fragmentation takes place in solution, solute–solvent interactions introduce additional pathways for radical recombination and secondary chemical transformations, adding complexity to the photodissociation dynamics in comparison to the dynamics observed in isolated systems. In this study, we investigate the ultrafast photodynamics of methyl *p*-tolyl sulfoxide (Metoso) in the 1–300 ps time range in dimethyl sulfoxide and ethanol solutions using ultrafast transient absorption spectroscopy. Density functional theory calculations have assisted in the interpretation of the experimental data. Upon UV irradiation, Metoso undergoes homolytic cleavage of one of the sulfur–carbon (S–CH_3_) bonds, leading to the formation of methyl and para tolylsulfinyl radicals. We explore the competition between geminate recombination, diffusional separation, and alternative reaction pathways. Our results provide insights into the early-stage photochemistry of alkyl–aryl sulfoxides, highlighting the role of solvent interactions influencing radical lifetimes, recombination, and reaction dynamics. These findings contribute to a deeper understanding of sulfoxide photoreactivity, which is relevant to medicinal chemistry and organic synthesis.

## Graphical abstract

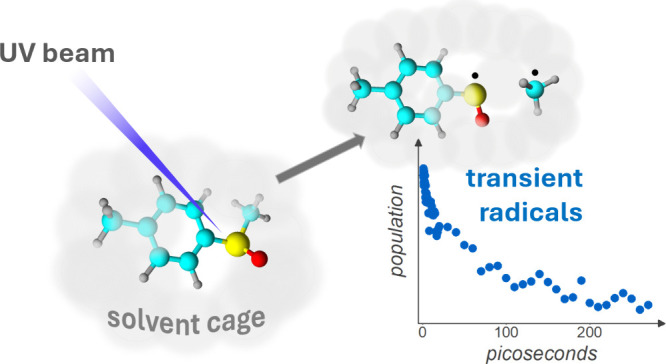

## 1 Introduction

The photogeneration and subsequent reactivity of radical species are central to many processes of biological and medicinal relevance. In medicinal chemistry, photochemically generated radicals have proven especially valuable for developing light-activated therapeutic strategies. At the same time, radicals are also key intermediates in atmospheric chemistry, combustion, and environmental processes, making their behavior a subject of both practical importance and fundamental interest.

A comprehensive understanding of radical formation and dynamics requires an examination of their behavior across different environments and conditions. The absence of numerous intermolecular interactions in the gas phase provides a simplified environment for observing and modeling fundamental mechanisms. The advent of molecular beam techniques, tunable fast and ultrafast pulsed lasers, and advancements in computational power and theoretical methods have allowed detailed insights into the dynamics of primary photofragmentation processes in isolated molecules^1–7^ and molecular clusters^8,9^ to be obtained.

While more complex, the condensed phase reflects the molecular environment of many practical and biological systems. However, solute–solvent interactions add layers of complexity to the investigation of photodissociation reactions. In solution, solute–solvent interactions can introduce relaxation or deactivation channels for excited molecules and their photofragments, with pathways often inaccessible in the gas phase. In particular, recombination processes, largely absent in the gas phase, are prevalent in solution, where photodissociation products in solution may recombine, sometimes after structural rearrangement, or react further with solvent molecules, giving rise to secondary chemical transformations.^7,10–14^

Recombination in solution is classified as geminate when the recombining species originate from the same parent molecule, even if the recombination product differs from the original structure. Geminate recombination is often subdivided into two types: primary and secondary.^15,16^ Primary geminate recombination occurs on ultrafast timescales, typically within picoseconds, where the radicals reunite almost immediately after bond cleavage. In contrast, secondary geminate recombination, also referred to as diffusion-limited geminate recombination, involves fragments initially separated by a thin layer of solvent molecules, forming the so-called contact pairs. These pairs recombine on longer timescales, ranging from tens to hundreds of picoseconds. Some radical pairs, however, escape the solvent cage, leading to recombination on diffusive timescales, often extending into the nanosecond range or longer.

Among the various classes of photoactive compounds, alkyl–aryl sulfoxides have been recognized for their ability to induce DNA strand cleavage under UV irradiation, a process initiated by radical formation.^17^ Sulfoxides represent a widely studied and utilized class of organo-sulfur compounds. They are involved in the metabolism of many biologically relevant sulfides^18–20^ and serve as versatile intermediates in organic synthesis.^21–25^ They contain the sulfinyl (S=O) functional group, with two organic substituents (R, R′) bound to the sulfur atom, which also has a free electron pair. The geometrical structure of sulfoxides (RR′S=O) is pyramidal, and they can be chiral if R ≠ R′ when the inversion barrier is sufficiently high not to be overcome by thermal energy.

The electronic and structural properties and the reactivity of aromatic sulfoxides, including their photochemistry, have been extensively investigated.^26–32^ In solution, upon exposure to UV radiation, the primary photoprocess of aryl–aryl and alkyl–aryl sulfoxides involves the homolytic cleavage of one of the carbon–sulfur bonds, resulting in the formation of an aryl sulfinyl and an alkyl­(aryl) radical pair.

The formation of aryl sulfinyl radicals (ArSO^•^) upon UV irradiation of aromatic sulfoxides in solution has been thoroughly documented.^27,29^ Their decay was investigated by nanosecond transient absorption spectroscopy, and it was shown that most alkyl- and arylsulfinyl radicals are transient at room temperature, as their extinction rate constants are close to the diffusion-controlled limit.^30^

It has also been reported that homolytic C–SO cleavage is followed by competition between diffusional separation of the radical pair to form free radicals, and geminate recombination to reform the sulfoxide and to form a sulfenic ester. Moreover, other reactions or secondary photoinduced reactions can occur,^29^ depending on the sulfoxide precursor structure and solvent, increasing the variety of possible products. Stereomutation of chiral aromatic sulfoxides due to UV irradiation is commonly attributed to random recombination of radicals, however, it has also been shown that, in some cases, aryl-sulfoxide photoracemization is accomplished by a noncleavage pathway, involving pyramidal inversion in the excited state.^31^ Moreover, in cryogenic matrices, the occurrence of isomerization to an oxathiyl radical PhOS^•^ has been reported.^33^

Given the complex chemistry of UV irradiated sulfoxides, we decided to investigate the dynamics of a relatively small sulfoxide, namely methyl *p*-tolyl sulfoxide (Metoso) in a solution of dimethyl sulfoxide (DMSO) or ethanol (EtOH) on the picosecond timescale utilizing ultrafast transient absorption spectroscopy and density functional theory (DFT) calculations. Scheme 1 reports the structure of Metoso, the fragment (Toso) and a reaction product identified in EtOH (Etoso) discussed in this paper. Our aim was to unravel the initial steps underlying both the formation and recombination of reactive intermediates.

**Scheme 1:**
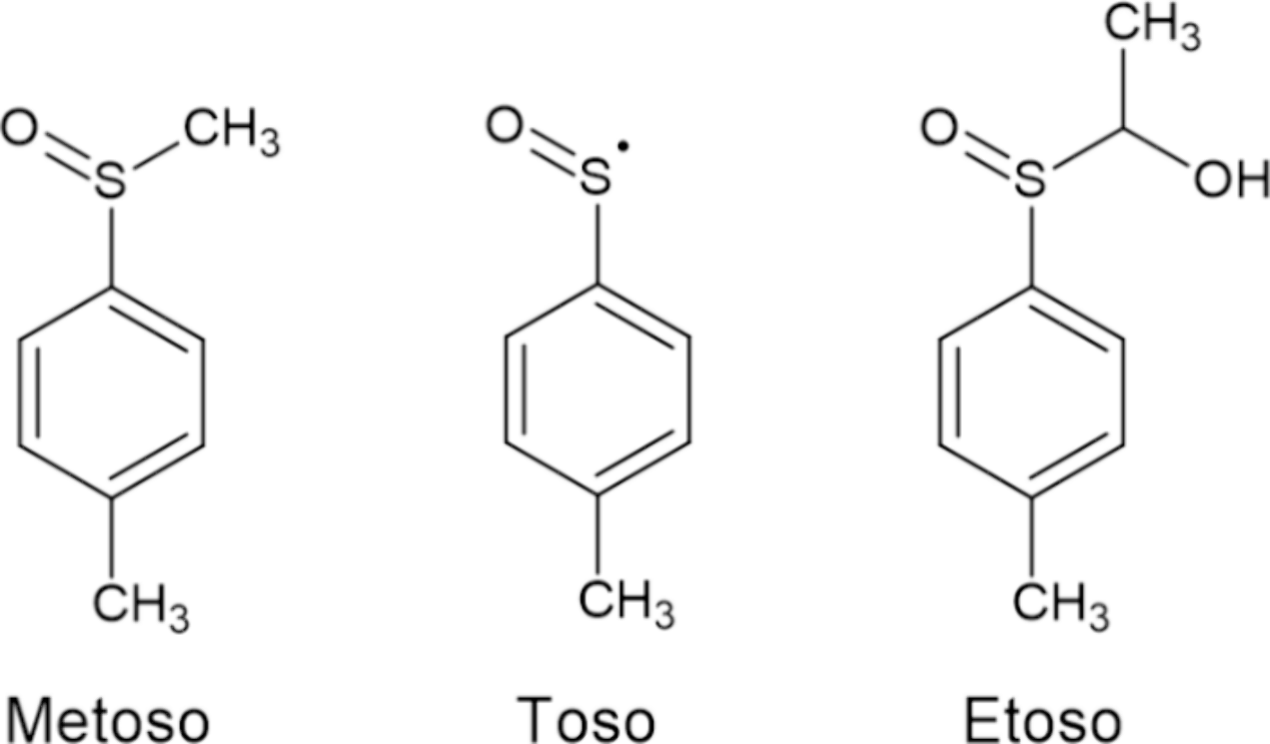
Molecular Structures of Methyl *p*-Tolyl Sulfoxide (Metoso), *p*-Tolyl Sulfinyl Radical (Toso) and *p*-Tolyl-1-hydroxyethyl Sulfoxide (Etoso)

## 2 Experimental and Theoretical Procedures

### 2.1 Experimental Section

Metoso (R enantiomer with 99% purity) was purchased from Sigma-Aldrich. Spectrophotometric grade EtOH and DMSO were used as received. Unless otherwise indicated, we used room temperature solutions at a concentration of 2 × 10^–2^ M in EtOH and DMSO, and the measurements were conducted using static fused silica cuvettes with an optical path length of 1 mm.

#### 2.1.1 Femtosecond Transient Absorption Spectroscopy

The transient absorption (TA) pump–probe experiments were performed using a laser system consisting of a regenerative amplifier seeded by a Ti:Sa oscillator, and generating pulses at 800 nm (1 kHz, 4 mJ, 35 fs). The output of an optical parametric amplifier was used for the pump pulse at 260 nm. The probe was generated by focusing 3 μJ of 800 nm light into a rotating CaF_2_ crystal, then collimating the white light supercontinuum generated, and subsequently focusing it onto the sample. The optical layout of the commercial TA spectrometer (Femto-Frame II, IB Photonics) consists of a split beam configuration in which 50% of the white light (350–700 nm) passes through the sample, while the remainder is used as a reference to account for pulse-to-pulse fluctuations in the white light generation. For the near-IR part of the spectrum, the white light was generated by focusing the 800 nm into a YAG crystal. The pump pulse is focused (circular spot diameter = 400 μm) onto the sample with a power density of 450 μJ cm^–2^. The probe pulse is smaller (approximately 150 μm) and is delayed in time with respect to the pump by varying the length of its optical path. All experiments were performed with linear polarization for both pump and probe pulses and the angle between the electric field vectors of the two was set to the magic angle to eliminate effects due to rotational dynamics. The instrument response function was measured to be approximately 50 fs. Further details of the setup can be found in a previous publication.^34^

Cuvettes with 50 μL of the samples were also irradiated under the 260 nm laser beam without focusing (spot size around 4 mm, power density 140 μJ cm^–2^) for 30 min.

#### 2.1.2 Steady-State Absorption and Photoluminescence Spectroscopy

The steady-state absorption spectra were recorded using a UV–vis spectrophotometer (Jasco V-630) in the wavelength range from 200 to 800 nm. The fresh and laser irradiated solutions 2 × 10^–2^ M in EtOH and DMSO were diluted 50-fold to measure the steady-state absorption spectra.

The photoluminescence spectra were acquired using a spectro-fluorophotometer (Shimadzu RF-6000) equipped with a Xe lamp. The excitation wavelength was set at 260 nm. Luminescence was collected in the wavelength range from 280 to 500 nm in order to cut off the excitation radiation and high-order bands and to reduce contributions from spurious signals. Photoluminescence spectra were acquired on as-prepared solutions and after irradiation under 260 nm lamp inside the spectro-fluorophotometer for 10 min.

#### 2.1.3 Gas Chromatography–Mass Spectrometry (GC–MS)

GC–MS analyses have been performed with a Shimadzu GCMS QP2010 Ultrasystem. GC–MS analysis of the nonirradiated EtOH solutions revealed a single predominant peak in the chromatogram with a retention time of 9.864 min, displaying ions in the mass spectrum characteristic of Metoso. The irradiated solution showed, alongside the peak at 9.864 min retention time (area 4.01%), an additional peak at a retention time of 11.075 min (area 8.31%), which, based on retention times and mass spectrum pattern, can be attributed to 4-methylbenzenesulfonic acid ethyl ester.

### 2.2 Theoretical Section

The computational study of the dissociation of Metoso into Toso and CH_3_ was carried out using the time-dependent DFT approach,^35^ based on the Becke Three-Parameter Hybrid Functional B3LYP.^36^ The basis set used for these calculations is the 6-31++G** with diffuse and polarization functions.^37^ Although this theoretical level is not particularly advanced, it was chosen because it reduces computational costs while still providing spectroscopic results in good agreement with experimental data, as will be discussed later. The effect of the solvent was simulated by employing the polarizable continuum model (PCM) using the integral equation formalism variant (IEFPCM).^38^ The minimum energy path (MEP) was constructed by optimizing all geometric degrees of freedom while scanning along the coordinate connecting the sulfur atom to the carbon atom of the methyl group. The scan step size was set to 0.1 Å.

The calculation of the absorption and photoluminescence spectra was performed using the following equation:

$$\varepsilon (\upsilon )=\sum \frac{N_{A}e^{2}\sqrt{\pi }}{1000\;\ln(10)m_{e}c^{2}}\frac{f_{i}}{\sigma }\;\exp\left[\frac{-(\upsilon -\upsilon_{i}{)}^{2}}{\sigma^{2}}\right]$$

where ε is expressed in units of L mol^–1^ cm^–1^, υ_*i*_ represents the transition energy in cm^–1^, *N*_A_ is Avogadro’s number (6.022 × 10^23^ mol^–1^), *c* is the speed of light (2.998 × 10^10^ cm s^–1^), and *m*_e_ is the electron mass (9.109 × 10^–31^ kg), *e* is the electron charge (1.602 × 10^–19^ C). σ accounts for the inhomogeneous broadening of electronic transitions in solution (here, we assume σ = 0.4 eV). *f_i_* represents the oscillator strength for the *i*th transition with energy *h*υ_*i*_. Moreover, the oscillator strength can be linked to transition dipole moment strength (*D_i_* ^2^):

$$f_{i}=\frac{8\pi m_{e}c\upsilon_{i}}{3e^{2}h}D_{i}^{2}$$

where *h* is Planck’s constant (6.626 × 10^–30^ kg cm^2^ s^–1^) and *D* the transition dipole moment in C cm.

We used the oscillator strengths calculated with Gaussian09 whenever possible (i.e., for absorption from the ground state) and numerically computed the transition dipole moment strength in cases where they were unavailable, such as when calculating the absorption or emission spectrum from an excited state. In this case the transition dipole moment strength has been calculated as

$$D_{i}^{2}=\sum {dx}_{ij}^{2}+{dy}_{ij}^{2}+{dz}_{ij}^{2}$$

where the sum for every initial state *i* is done for all the state *j* within the energy window considered. The calculation of the transition dipole moment strength is done by computing separately their Cartesian components:

$$ds_{ij}=\langle \Psi_{i}\lfloor e\cdot s\rfloor \Psi_{j}\rangle ,s=x,y,z$$

These Cartesian components of the transition dipole moment strength were evaluated as a sum of integrals over molecular orbitals (ψ) over which the TD wave functions (Ψ) is expanded:

$$\langle \Psi_{i}\lfloor e\cdot s\rfloor \Psi_{j}\rangle =\sum c_{k}c_{t}\langle \psi_{k}\lfloor e\cdot s\rfloor \psi_{t}\rangle ,\;\;s=x,\;y,\;z$$

In particular the TD wave functions of the initial and final states involved in the excitations were used with threshold of 0.1 for the coefficients (*c*) in the CI expansion. The numerical calculations of the integrals in the right side of the above equation were performed by using a cubic box of 40 Å per side, using a grid of one million points each.

## 3 Results

### 3.1 Potential Energy Curves

The vertical transition of Metoso from the neutral ground state S_0_ to the electronically excited state S_1_ is calculated to be 4.81 eV in DMSO (258 nm) and 4.86 eV in EtOH (255 nm). This transition is illustrated by the purple arrow in Figure 1, with the thick black bars representing the S_0_ and S_1_ states in the optimized geometry of the ground state.

**Figure 1:**
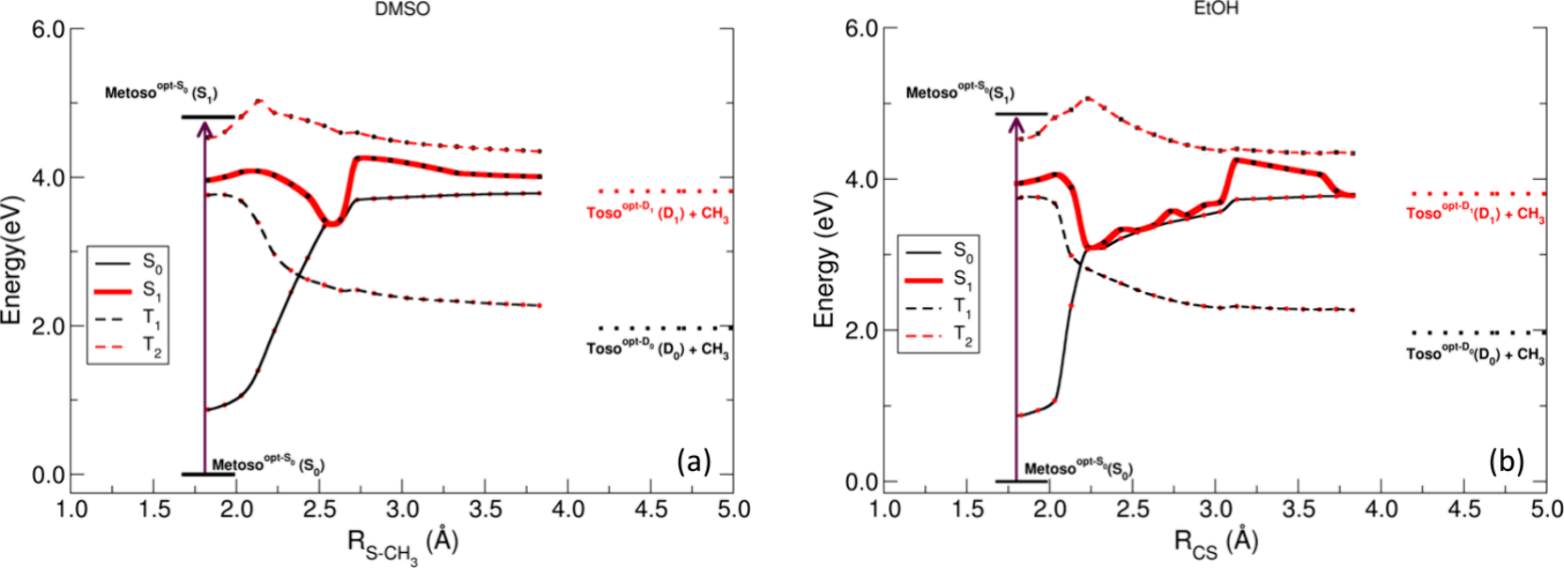
Minimum energy path for the dissociation of Metoso in its first excited electronic state S_1_ along the S–CH_3_ bond distance (red solid bold curve) in DMSO (a) and in EtOH (b) solvents, calculated at the TD-B3LYP/6-31++g** level. The other curves refer to singlet and triplet electronic states at the geometries of the MEP of S_1_ state. On the right of both panels are reported the energy levels of the methyl plus Toso radical. The purple arrows illustrate the vertical transition of Metoso from the neutral ground state S_0_ to the electronically excited state S_1_, with the thick black bars representing the S_0_ and S_1_ states in the optimized geometry of the ground state. See the text for further details.

The energy curves are shown as a function of the coordinate linking sulfur to the carbon of the methyl group. Specifically, the thick red curve represents the minimum energy path along the dissociative S–CH_3_ coordinate, where all other geometric parameters are optimized. The black curves correspond to the S_0_ state (solid curve) and T_1_ triplet state (dashed curve) of Metoso at the S_1_ optimized geometries. The same scheme is adopted for the red dashed curve T_2_ triplet state. A complete view, including the higher energy excited states, is reported in Figure S1 of the Supporting Information.

In both solvents, following the vertical excitation S_1_ ← S_0_, the system is expected to initially relax toward the S_1_ excited state, which is approximately 0.85 eV lower in the case of DMSO solvent and about 0.92 eV lower in the case of EtOH solvent. This geometric relaxation process initially involves a simultaneous excitation of vibrational levels, which can only later thermalize with the solvent.

In both solvents, the elongation of the *S*-methyl bond initially encounters a dissociation barrier that is relatively small (0.13 eV for DMSO and 0.14 eV for EtOH). This barrier is followed by a slightly deeper energy minimum in EtOH (−0.92 eV relative to the barrier) compared to DMSO (−0.85 eV). It is worth noting that both the position of the barrier and the position of the second absolute minimum are quite similar for EtOH and DMSO; the barrier is localized at *R*_S–C_ = 2.05 Å for EtOH and 2.10 Å for DMSO, while the absolute minimum is at *R*_S–C_ = 2.25 Å for EtOH and 2.58 Å for DMSO. This suggests that the elongation of the S–C bond is only slightly more favored in DMSO than in EtOH.

The differences between the two solvents become more pronounced when considering the position of the second, higher barrier leading to final dissociation. In the case of EtOH, the barrier is located at a greater bond distance (3.13 Å) compared to DMSO (2.77 Å).

A second important difference between the two solvents lies in the way the S_1_ and S_0_ curves approach each other in the region of maximum contact, which is located between the two barriers. In the case of EtOH, there is a broad contact area between the first two singlet electronic states, whereas in DMSO, the contact between S_0_ and S_1_ is limited to a rather narrow region around the minimum, located at *R*_S–C_ ≈ 2.6 Å.

It should also be noted that in both solvents during the dissociation along the coordinate S–C, there is no crossing between S_1_ and the ground-state triplet curve T_1_. The other electronic states do not appear to participate in the dissociation dynamics, as they are all at energy levels too high to be considered (see Figure S1). The only state potentially involved in the de-excitation process is the first excited triplet electronic state T_2_ (thin red dashed line in Figure 1), but it cannot be populated during the vertical pump transition since it is forbidden due to spin symmetry. On the right side of both panels of Figure 1, the energy levels related to the dissociation of Metoso into Toso + CH_3_ are shown. The two dotted bars represent the methyl group in its relaxed geometry in the fundamental doublet state, while Toso is in the equilibrium geometries of the doublet states D_0_ (black) and D_1_ (red).

### 3.2 Calculated Absorption and Emission Spectra

The absorption and photoluminescence spectra of Metoso in the S_1_ excited state were calculated at the TD-B3LYP/6-31++G** theoretical level and are shown as red lines in Figure 2a (DMSO solvent) and Figure 2b (EtOH solvent).

**Figure 2:**
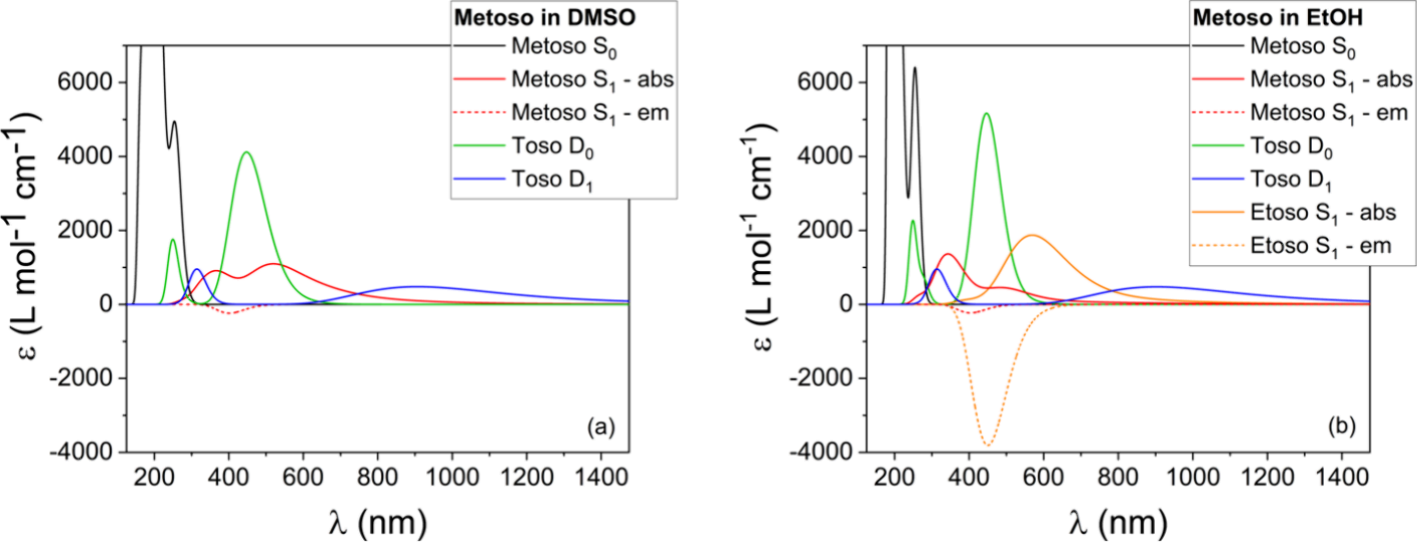
Calculated absorption spectra in DMSO (a) and EtOH (b) solvent of Metoso S_0_ (black), Metoso S_1_ (red), Toso D_0_ (green), Toso D_1_ (blue), and Etoso S_1_ (orange). For Metoso S_1_ and Etoso S_1_, the photoluminescence spectra are also reported as negative signals in red and orange dotted lines, respectively.

In the case of the DMSO solvent, the simulations for Metoso S_1_ predict a broad absorption feature in the range 300–700 nm and a weak photoluminescence signal around 400 nm (see Figure 2a). The calculated absorption spectra in the range 200–1500 nm of Toso radical in the ground D_0_ (green line) and excited D_1_ (blue line) states are also shown. In this range, Toso D_0_ presents two features with maxima at 250 and 450 nm, Toso D_1_ has absorption bands around 310 nm and bands extending to the near-IR region. Simulations for Toso D_1_ predict emission features with negligible oscillator strength.

Figure 2b shows the calculated absorption and photoluminescence spectra in EtOH for Metoso S_1_ (red curves), Toso D_0_ (green curve) and Toso D_1_ (blue curve). Absorption features are predicted for Metoso S_1_ in the range 300–600 nm, around 250 and 445 nm for Toso D_0_, while Toso D_1_ presents an absorption band at 315 nm and a broad feature in the 650–1400 nm region. Simulations predict emission features with negligible oscillator strength for Toso D_1_ and very weak for Metoso S_1_ around 400 nm.

Figure 2b also shows the computed absorption (orange solid line) and photoluminescence (orange dashed line) spectra for the species Etoso (see Scheme 1) in its excited S_1_ state, which will be introduced and discussed in the next sections. As can be seen, this species is predicted to absorb around 450–750 nm and to strongly emit around 450 nm.

### 3.3 Steady-State Absorption and Photoluminescence Spectra

Figure 3a,b shows the steady-state absorption spectra (black lines) of a 4.0 × 10^–4^ M solution of Metoso in DMSO and EtOH, respectively. DMSO has a high extinction in the UV range, which prevents the measurement of the signals of Metoso in DMSO at wavelengths lower than 250 nm (see gray area in Figure 3a): the spectroscopic feature of Metoso appears as a large absorption signal below 280 nm increasing toward short wavelengths, but the position of the maximum cannot be assessed. In the case of EtOH, a relative maximum at 237 nm is observable in the UV spectrum with a tail extending to about 280 nm.

**Figure 3:**
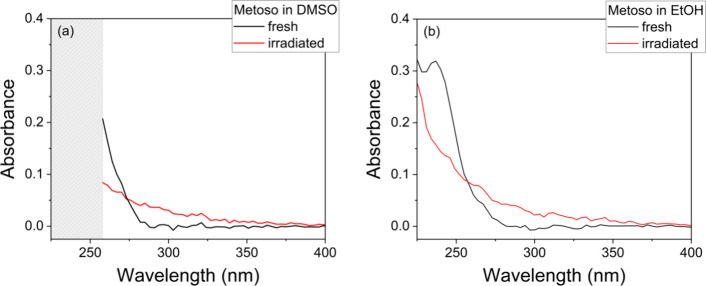
Steady-state absorption spectra of fresh (black line) and irradiated at 260 nm (red line) 4.0 × 10^–4^ M solution of Metoso in (a) DMSO and (b) EtOH. The absorbance values are calculated using the formula *A* = log_10_(transmittance_solvent_/transmittance_sample_).

After laser irradiation (as described in Section 2.1) of the samples, both the DMSO and EtOH solutions showed a decrease of the Metoso signal and the appearance of a broad absorption up to 350 nm, suggesting that a significant photodegradation of the sample has occurred (see red lines in Figure 3a,b).

Photoluminescence spectra are reported in Figure 4 for Metoso in DMSO and EtOH. As can be seen, no appreciable signals are detected in DMSO, neither for the fresh (black dotted line) nor for the irradiated (red dotted line) sample, apart from weak broad background features. For the fresh solution, this agrees with simulations, which predict only emission features with negligible and very weak oscillator strength for Metoso S_1_ and Toso D_1_ (see Figure 2a). Furthermore, we can conclude that in DMSO the photodegradation products, which may be present in the irradiated solution, are nonfluorescent.

**Figure 4:**
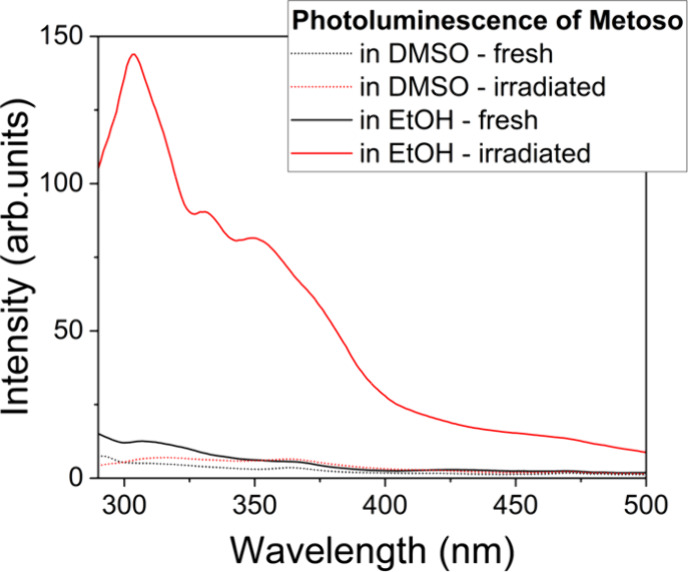
Photoluminescence spectra upon excitation at 260 nm of fresh (black lines) and irradiated (red lines) 2.0 × 10^–2^ M solution of Metoso in DMSO (dot lines) and EtOH (solid lines).

In the case of EtOH solution, an intense fluorescence feature is observed in the irradiated solution of Metoso, which is not present in the fresh solution (see red and black solid lines in Figure 4). This finding suggests that upon UV irradiation some new species, which strongly emit in the 300–400 nm range, is/are produced in the EtOH solution.

### 3.4 Transient Absorption Spectra

TA spectra were recorded at pump–probe time delays between −1 and 300 ps over the 350–700 and 970–1030 nm spectral regions following excitation at 260 nm. Figure 5a,b shows the TA spectra at selected time delays for 2.0 × 10^–2^ M solution of Metoso in DMSO excited at 260 nm. Positive transient absorbance (Δ*A*) signals are measured across all the UV–vis and NIR spectral ranges, suggesting a dominant contribution of photoinduced absorption of the probe from photoexcited states. Comparing the TA spectra at increasing time delays in Figure 5a, it can be seen that a rapid decay over a few picoseconds is followed by a slower component lasting several hundred picoseconds or more. However, the temporal behavior varies across the different spectral visible regions, with the slower decay becoming progressively shorter moving toward the higher-wavelength region as shown in Figure S2 of the Supporting Information. This behavior suggests the presence of different species contributing to the TA spectra in the visible region. In the NIR spectral range the slower decay is even shorter with respect to the visible region and appears to decay homogeneously across the NIR spectral range investigated (Figure 5b).

**Figure 5:**
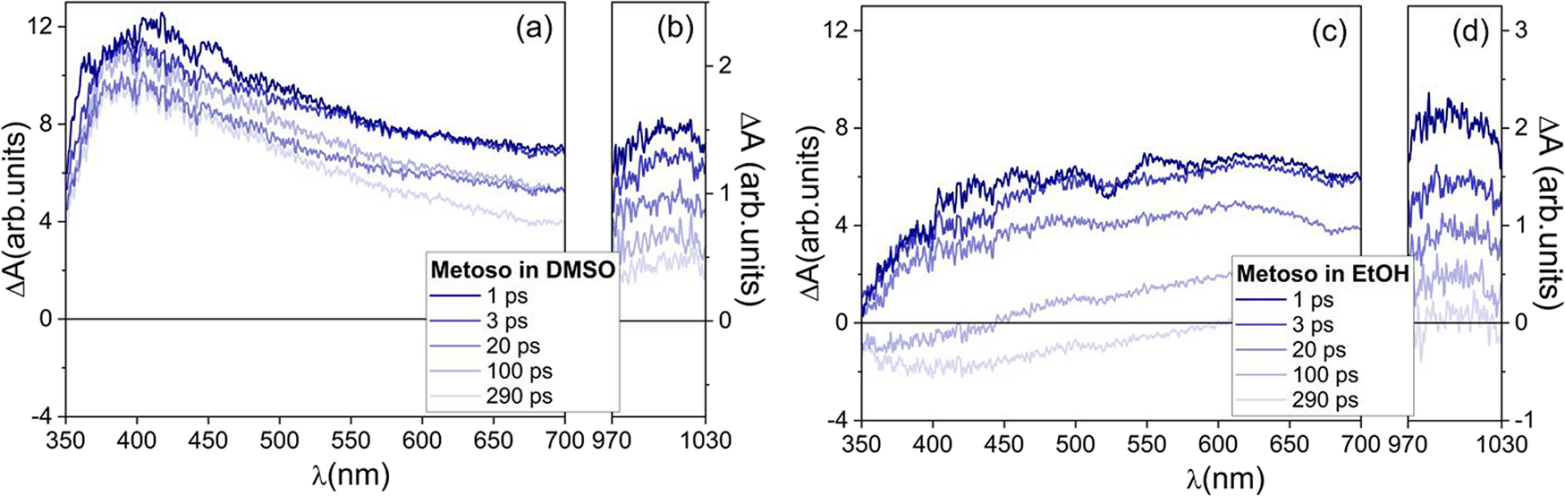
TA spectra at selected time delays for 2.0 × 10^–2^ M solution of Metoso in DMSO (a, b) and EtOH (c, d) excited at 260 nm. Data at time delays below 1 ps were excluded due to interference from cross-phase effects.

In Figure 5c,d the TA spectra at selected time delays for 2.0 × 10^–2^ M solutions of Metoso in EtOH, excited at 260 nm are shown. A marked difference with the spectra measured in DMSO is observed. Photoinduced absorption bands are prevalent at short time delays (1–70 ps) in the whole investigated region and are relatively weaker in the 350–450 nm region in comparison with DMSO solutions. At time delays larger than 50 ps, a strong signal with negative Δ*A* is measured in the 350–500 nm region. Negative signals indicate either a transient bleaching or stimulated emission. Since the Metoso solution has no steady-state absorption to bleach in this spectral range we conclude that the negative signal is due to the stimulated emission bands. The temporal behavior varies across the range 350–700 nm, suggesting the presence of different species contributing to the TA in the visible region.

In order to identify the transient species that contribute to the TA spectra of Figure 5, the calculated absorption and photoluminescence spectra of Figure 2 have been examined. Besides absorption bands due to Metoso in the S_1_ excited state, other species have to be considered, that may have been generated in the first picosecond after pump excitation. We considered that the Toso radical (see Scheme 1) resulting from the S–CH_3_ homolytic cleavage, both in its ground D_0_ and in its excited D_1_ state may contribute to the measured TA spectra. In particular, the Toso radical in its excited D_1_ state is predicted to have photoinduced absorption bands extending to the near-IR region, as can be seen from Figure 2a,b, and this is true in both DMSO and EtOH. We have also examined the absorption spectra of plausible ground state radicals and recombination products which could be easily produced from fragmentation and/or recombination of the Toso and CH_3_ radicals (such as some of those shown in Table S1 of the Supporting Information) and none of them has absorption energies around 1000 nm. We also considered a possible intersystem crossing to the T_1_ triplet state. Geometry optimization of the triplet state in its T_1_ electronic configuration did not yield a local minimum on the corresponding potential energy surface. Instead, the T_1_ state turned out to be dissociative, resulting in the formation of Toso D_0_.

We thus hypothesize that the positive signals in Figure 5b,d can be attributed to the photoinduced absorption of the Toso radical in the D_1_ excited state. Following the temporal profile of the NIR signals we obtained information on the Toso D_1_ dynamics in both solvents: a fast decay on the timescale of few picoseconds is followed by a slow decay on the hundreds of picoseconds timescale, i.e. with time constants of τ_1_ = 5.0 ± 1 ps and τ_2_ = 300 ± 20 ps in DMSO and τ_1_ = 1.5 ± 0.2 ps and τ_2_ = 166 ± 18 ps in EtOH. The biexponential fits of the experimental data are reported in Figure 6.

**Figure 6:**
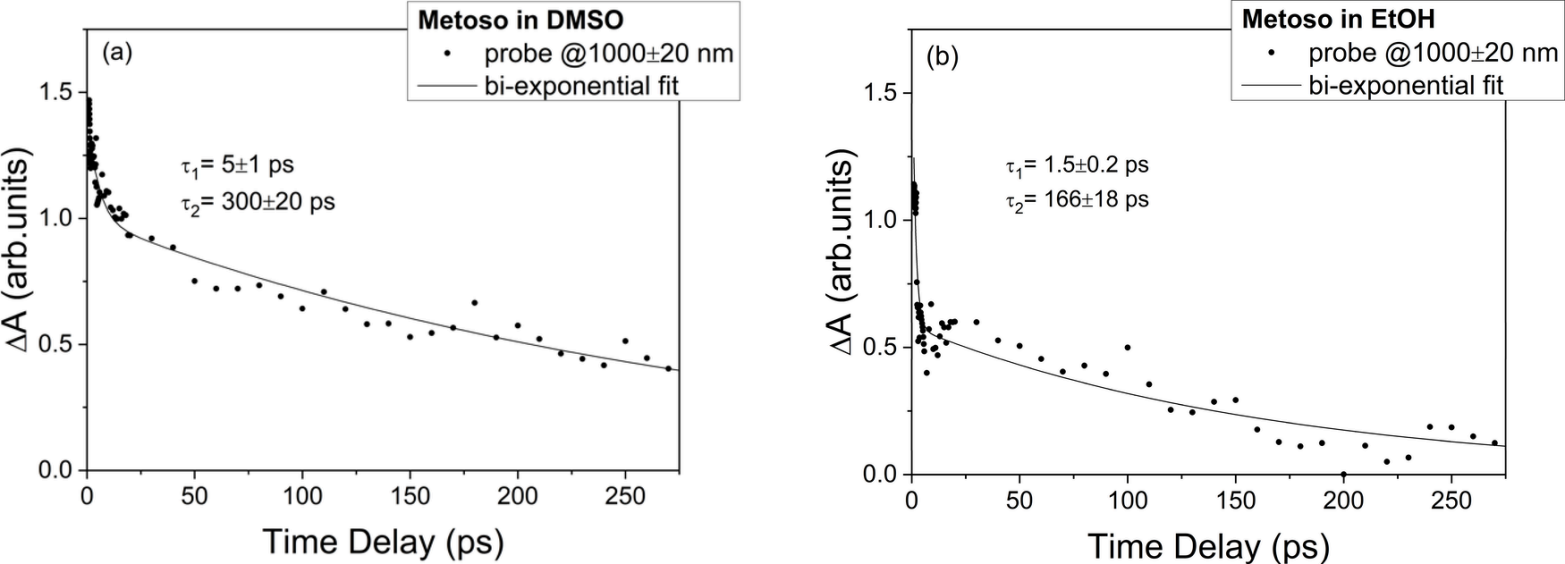
Temporal profiles integrated in the NIR region and biexponential fits for 2.0 × 10^–2^ M solution of Metoso in DMSO (a) and ethanol (b) excited at 260 nm.

In contrast, in the 350–700 nm spectral range, a spectral overlap may occur due to contributions to the TA spectra from several different species which are predicted to absorb and emit in this region (see Figure 2a,b). In DMSO the species that may contribute to the transient absorption in the visible region are Metoso in the S_1_ state and the Toso radical in both D_0_ and D_1_ state. To disentangle the contribution of each species is, however, not straightforward.

In the case of the TA spectra in EtOH (Figure 5c), the negative signal in the 350–600 nm range for time delays greater than 50 ps stems from stimulated emission bands from an excited species which is formed following excitation and dissociation of Metoso in EtOH. These bands cannot be attributed to Metoso S_1_ or Toso D_1_ since calculations predict negligible or very weak emission, respectively, for Metoso S_1_, and Toso D_1_ in the 400–500 nm range.

We therefore considered different products that could be formed by the recombination of the Toso and CH_3_ radicals, such as those reported in Table S1 of the Supporting Information. The computed transition dipole moments for almost all these species are negligible in the wavelength range 450–500 nm and therefore most of them cannot account for the observed negative Δ*A* signals at *t* > 50 ps.

The understanding of this stimulated emission takes on a new dimension when we consider the possibility of a hydrogen abstraction reaction from the EtOH solvent by the methyl radical,^39,40^ forming methane and the 1-hydroxyethyl radical, which can recombine with Toso D_1_ to produce the excited state of *p*-tolyl-1-hydroxyethyl sulfoxide (see Scheme 1), i.e. Etoso S_1_. As shown in Figure 2b, the photoluminescence spectrum of Etoso S_1_ is relatively intense around 450 nm. Etoso in the S_1_ state is a transient species, which however provides further evidence that the Toso radical exists as an excited species in solution in the observed time window, as the Etoso product in an excited state could not be formed otherwise.

## 4 Discussion

It has been previously reported that the photodissociation of aromatic sulfur-containing compounds in solution, specifically aromatic thiols, thioethers and disulfides, occurs on the subpicosecond timescale. The process predominantly forms aromatic thiyl radicals in the ground state, as well as sandwich-type π-stacked radical dimers.^10–14,41^ In contrast, excitation of an aromatic sulfoxide in solution, such as Metoso, reveals a TA signal that we have tentatively associated with the presence of the Toso sulfinyl radical in the D_1_ excited state.

As shown in the potential energy curves in Figure 1, excitation of Metoso at 260 nm produces vibrationally excited Metoso in the S_1_ state. Based on this, we infer that part of the vibrationally unrelaxed Metoso in the S_1_ state undergoes ultrafast (≪1 ps) dissociation, while the remainder moves toward a minimum on the S_1_ potential energy surface and may either relax to the S_0_ electronic ground state through internal conversion (IC) or undergo further dissociation. Within the timescale of our experiment, several pathways are possible and those in DMSO solution will be described first. Figure 7 presents a simplified model for the dynamics occurring in DMSO solutions.

**Figure 7:**
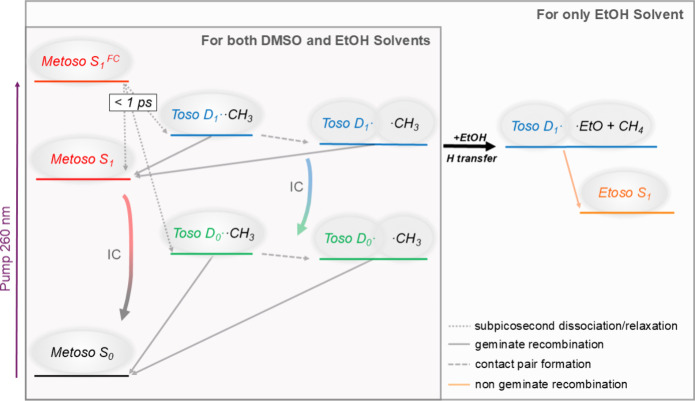
Simplified representation of the dynamics occurring upon photoexcitation at 260 nm of Metoso in DMSO and EtOH solutions.

The transient excited Toso D_1_ radical can recombine geminately. Typically, up to a few picoseconds, the radicals formed are confined within the solvent cage, where a fraction of them can readily recombine. As time progresses, they begin to move apart and rotate, leading to a lower recombination efficiency. The phenomenological timescales, τ_1_ = 5.0 ± 1 ps and τ_2_ = 300 ± 20 ps qualitatively match lifetimes assigned to primary and secondary geminate recombination of aromatic sulfur radicals in solution,^12^ yet relaxation of the Toso D_1_ radical to the Toso D_0_ ground state most likely contributes to the observed kinetics.

In the visible region, especially in the lower wavelength region, after a fast decay of few picoseconds, the signal appears to persist well beyond the 300 ps range we have examined in the TA spectra. Since the decay in the 1000 nm region, attributed to Toso D_1_, is faster, the long decay may be attributed either to the nongeminate recombination of the Toso D_0_ and CH_3_ radicals or to the decay of Metoso S_1_.

Although the Metoso molecule in its electronic ground state is not observable in the 300–700 nm range of the TA spectra, the regeneration of ground-state molecules is essential. This is evident from the fact that the probed solution continues to exhibit the same behavior regarding TA spectra, even after measurement periods lasting more than 1 h. The reformation of ground-state Metoso can occur through two possible pathways: recombination (geminate or bimolecular) of ground-state Toso D_0_ and CH_3_ radicals or internal conversion from the Metoso S_1_ state. As shown in Figure 1, a crossing between the S_1_ and S_0_ electronic states of Metoso along the S–CH_3_ stretching coordinate is theoretically predicted. Metoso S_1_ may therefore decay by relaxation processes such as IC and solvent quenching.

It should be noted that in this model, we did not consider ultrafast reactions in DMSO solutions, as there is no clear evidence of reaction products on the 300 ps timescale. However, the steady-state UV spectrum of a solution irradiated for 30 min on a significant fraction of the sample with a fluence of 140 μJ cm^–2^ (see Figure 3a), shows that other products are formed which absorb at around 300 nm. This suggests that some radicals may recombine, forming products that differ from the original structure. It is possible that this occurs on a timescale beyond the 300 ps range we have examined in the TA spectra, yet a small fraction of radicals could undergo this process on a shorter timescale. To disentangle a possible contribution to the TA spectra deriving from a different recombination product is, however, not straightforward.

It should be noted that, unlike DMSO solution, we observe some degradation of Metoso in EtOH solution even during the acquisition of the TA spectra. For this reason, we have used freshly prepared solutions to minimize the impact of degradation. As found for DMSO solutions, steady-state UV spectra revealed that, after prolonged irradiation, Metoso has undergone extensive degradation, and other products are formed which absorb around 300 nm (see Figure 3b).

Further confirmation that a reaction occurs between the Toso and deprotonated EtOH radicals comes from GC–MS analysis and from steady-state fluorescence spectra of the nonirradiated and irradiated EtOH solutions. A GC–MS analysis of the irradiated solution exhibited, in addition to features due to Metoso, other peaks attributed to 4-methylbenzenesulfonic acid ethyl ester which were not detected in the nonirradiated solution. This species is probably formed by slower secondary reactions of Etoso, such as oxidation in air and rearrangement of the CH_3_CHOH group. The observed product aligns with the proposed mechanism on the ultrafast timescale, which takes into consideration the participation of solvent molecules. Furthermore, the steady-state photoluminescence spectrum of the irradiated solutions of Metoso in EtOH (Figure 4) displays a fluorescence signal within the 300–400 nm spectral range which is not present in the nonirradiated solution. The emission range is consistent with the emission of 4-methylbenzenesulfonic acid ethyl ester, the species also found by GC–MS analysis.

Figure 7 presents a simplified scheme for the dynamics occurring in EtOH solution. As in the case of the DMSO solvent, Metoso S_1_ may undergo relaxation processes such as IC and solvent quenching. An initial fast decay is observed for the Toso D_1_ radical (τ_1_ = 1.5 ± 0.2 ps), which can be related to fast geminate recombination within the solvent cage. Indeed, the reaction of Toso D_1_ with the solvent can have important effects on the dynamics of the processes. At time delays longer than 50 ps, a strong signal with negative Δ*A* signal in the 350–500 nm region is observed, which we propose to attribute to the emission of Etoso S_1_. As can be seen from Figure 7, this product can be formed through hydrogen transfer from a solvent molecule to the methyl radical, followed by recombination of the 1-hydroxyethyl radical with Toso D_1_. The reaction involving the methyl radical, ethanol and the Toso D_1_ radical producing Etoso S_1_ and CH_4_ is exothermic by 0.3 eV (at B3LYP/6-31++G** level of theory) and therefore, from a purely thermodynamic point of view, this fast reaction is energetically possible.

Moreover, the τ_2_ = 166 ± 18 ps time constant obtained for the long time-scale decay of Toso D_1_ radical is compatible with the observation of the stimulated emission signal at time delays larger than 50 ps, confirming the presence and the reactive loss of Toso D_1_. A reaction between ground-state Toso D_0_ radical and the 1-hydroxyethyl radical could also occur, and this would shorten also the lifetime of the Toso D_0_ radical in EtOH, although its value cannot be evaluated.

In EtOH solution, due to the lower viscosity of the solvent with respect to DMSO, diffusion-limited geminate recombination processes are expected to be faster, however probably significantly slower than the reaction of CH_3_ with EtOH molecules. If this is the case, secondary geminate recombination and the potential reformation of Metoso S_1_ and Metoso S_0_ are inhibited due to the rapid disappearance of CH_3_.

## 5 Conclusions

The photochemical reaction dynamics of the Toso radical, produced by 260 nm photolysis of Metoso is examined in the 1–300 ps time range in DMSO and EtOH solutions. In order to explain our results, we considered the formation of the Toso radical both in the ground (D_0_) and excited (D_1_) states. Previous works on radicals formed following excitation of aromatic thiols and disulfides consider only the ground state of the formed thiyl radicals. In the case of aromatic sulfoxides, we show that considering only the ground-state Toso D_0_ radical would not be sufficient to describe the transient spectra measured. Particularly strong evidence for the involvement of the excited Toso radical (D_1_) in the dynamics can be found in the observed transient photoinduced absorbance in the IR region of the spectrum (around 1000 nm), which, according to the theoretical calculations performed, cannot be assigned to any of the other species considered.

In DMSO the photolytically generated Toso D_1_ radical decays through geminate recombination to the parent molecule and competitive relaxation to the D_0_ ground state. A fraction of the Toso D_0_ radicals persists for longer times than those probed by transient spectroscopy and recombines, forming products that differ from the original structure and then undergo further complex reactions, as demonstrated by UV absorption spectra of irradiated samples.

In EtOH solution, an additional reaction of the Toso D_1_ radical is deduced from the rise of a stimulated emission signal in the transient spectra attributed to the Etoso S_1_ product. This arises due to the reaction of Toso D_1_ with the 1-hydroxyethyl radical, formed through hydrogen transfer from an EtOH molecule to the methyl radical. The formation of this product is further evidence of the central role of the excited Toso D_1_ radical in the photoreactivity of Metoso. Photoluminescence measurements confirm the presence of reaction products in EtOH that were not present in the DMSO solution.

## Supplementary Material



## Notes

Table of the predicted emission wavelengths of the species potentially formed upon fragmentation or recombination of the Toso and CH_3_ radicals, extended description of the electronic landscape associated with the S_1_ dissociation of Metoso, including the higher-lying excited-state levels of both Metoso and the Toso + CH_3_ radical pair, and time-resolved profiles integrated over selected visible and infrared regions for Metoso in DMSO excited at 260 nm (PDF)
