# Supplementary material for: Ultrafast Photodissociation and Recombination Dynamics of Methyl p‑Tolyl Sulfoxide in Solution: Insights from Transient Absorption Spectroscopy and Computational Studies
Source: ACS Omega. 2026 May 25;11(22):32086–95. doi: 10.1021/acsomega.5c11932 (PMC13261449; doi:10.1021/acsomega.5c11932)
Supplement: Supplementary file 1 [file ao5c11932_si_001.pdf]

Supporting Information

Ultrafast Photodissociation and Recombination Dynamics of Methyl p-Tolyl Sulfoxide in Solution: Insights from Transient Absorption Spectroscopy and Computational Studies.

Alessandra Paladini, [a] Patrick O'Keeffe, [a] Francesco Toschi, [b] Daniele Catone, [b] Stefano Turchini, [b] Giuseppe Ammirati, [b] Pierluca Galloni, [c] Mauro Satta,[d] and Susanna Piccirillo [b,c]**

[a] *CNR-Istituto di Struttura della Materia (CNR-ISM), EuroFEL Support Laboratory (EFSL), Via Salaria km 29,300, 00015 Monterotondo Scalo (RM), Italy*

[b] *CNR-Istituto di Struttura della Materia (CNR-ISM), EuroFEL Support Laboratory (EFSL), Via del Fosso del Cavaliere 100, 00133 Roma, Italy*

[c] *Dipartimento di Scienze e Tecnologie Chimiche, Università di Roma "Tor Vergata", Via della Ricerca Scientifica, 00133 Rome, Italy*

[d] *CNR-Istituto per lo studio dei Materiali Nanostrutturati (CNR-ISMN), Dip.to di Chimica, Università Sapienza di Roma, P. le Aldo Moro 5, Roma, 00185, Italy*

Table S1. Structures and predicted emission wavelengths of the different reactive products that could be formed by the fragmentation and/or recombination of the Toso and CH_3 radicals.

**Structure**	**Emission (nm)**
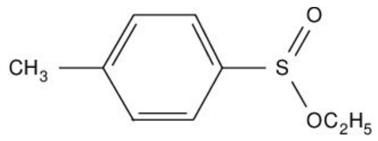	296
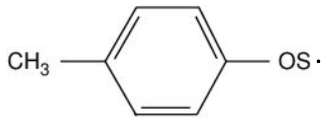	1418
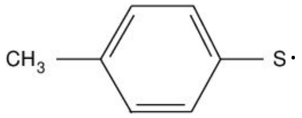	2366
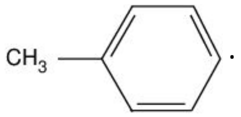	843
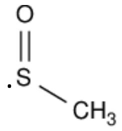	827
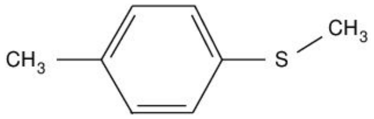	334
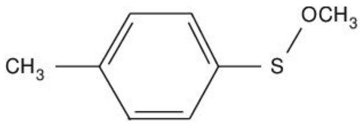	> 821
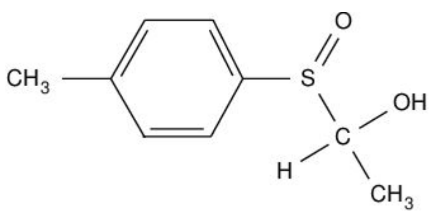	430

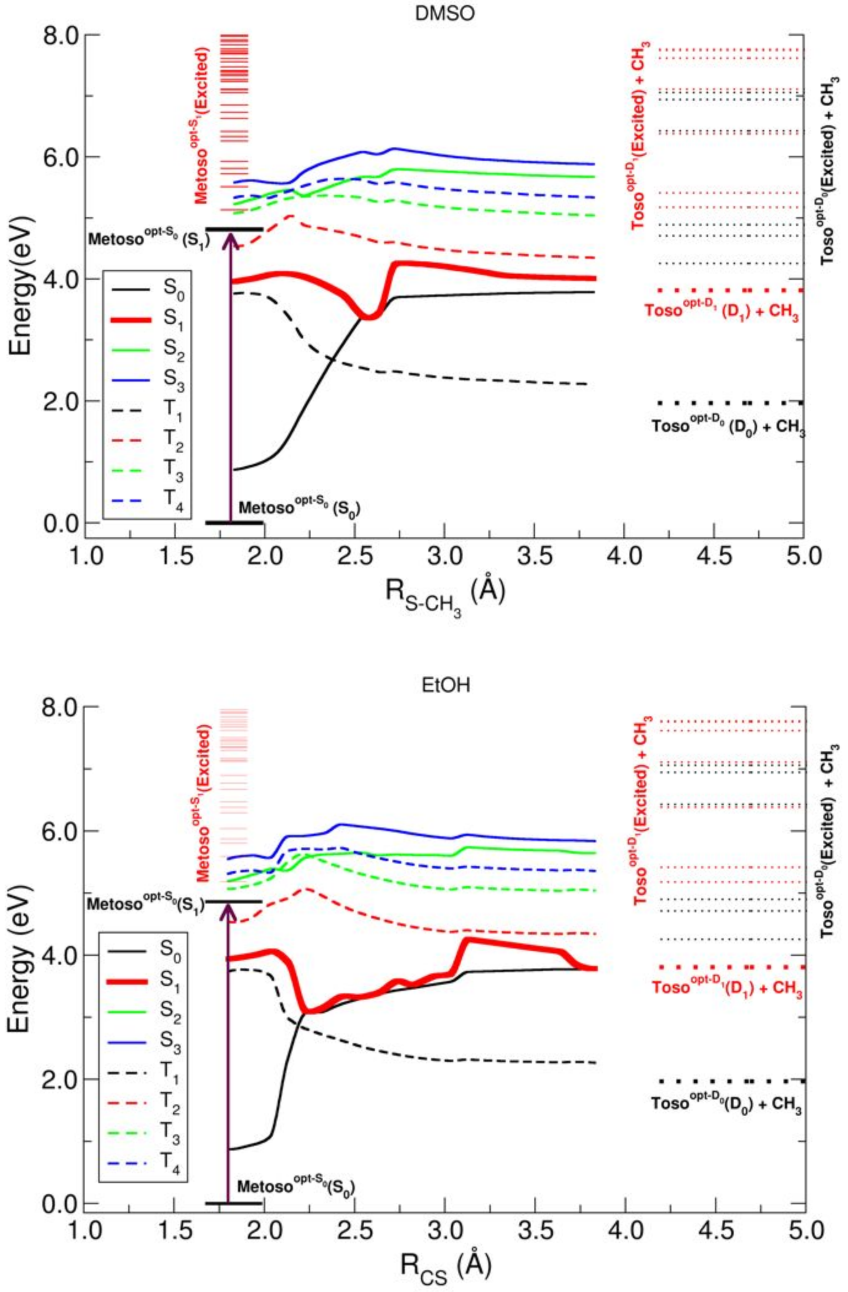

Figure S1. Minimum Energy Path for the dissociation of Metoso in its first excited electronic state S_1 along the S–CH_3 bond distance (red solid bold curve) in DMSO (top) and in ETOH (bottom) solvents, calculated at the TD-B3LYP/6-31++g** level. All the other curves refer to singlet and triplet electronic states at the geometries of the MEP of S_1 state. On the left of both panels are reported the energy levels of Metoso in the minimum energy geometry of the S_1 state. On the right of both panels are reported the energy levels of the methyl plus Toso radical (thick dotted bars). The thin black dotted bars correspond to vertical excitations from the equilibrium geometry of Toso D_0, while the thin red dotted bars represent vertical excitations from the equilibrium geometry of Toso D_1.

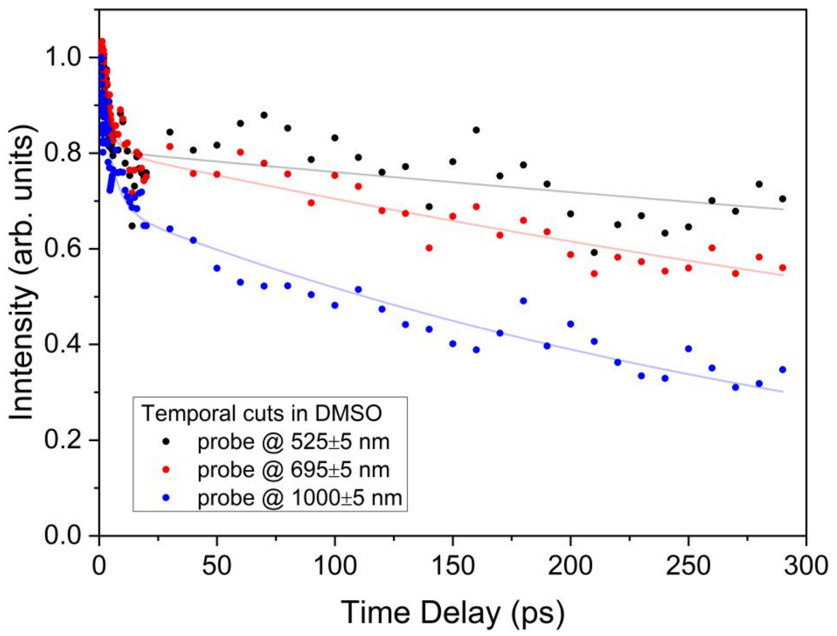

Figure S2. Temporal profiles integrated in the 525±5 nm (black dots), 695±5 nm (red dots), and 1000±5 nm (blue dots) regions for 2.0 10^{-2} M solution of Metoso in DMSO excited at 260 nm. To guide the eye, their bi-exponential fits are shown in solid lines.
